# Burden of Neonatal Surgical Conditions in Northern Ghana

**DOI:** 10.1007/s00268-019-05210-9

**Published:** 2019-10-03

**Authors:** Alhassan Abdul-Mumin, Theophilus T. K. Anyomih, Sheila A. Owusu, Naomi Wright, Janae Decker, Kelli Niemeier, Gabriel Benavidez, Francis A. Abantanga, Emily R. Smith, Stephen Tabiri

**Affiliations:** 1 School of Medicine and Health Sciences, University for Development Studies, Tamale, Ghana; 2 Tamale Teaching Hospital, Salaga Road, Tamale, Ghana; 3 King’s Centre for Global Health and Health Partnerships, School of Population Health and Environmental Sciences, King’s College London, London, SE5 9RJ UK; 4 Department of Public Health, Baylor University, 1301 S University Parks Dr, Waco, TX 76706 USA; 5 Duke Global Health Institute, Duke University, 310 Trent Dr, Durham, NC 27710 USA

## Abstract

**Background:**

Congenital anomalies have risen to become the fifth leading cause of under-five mortality globally. The majority of deaths and disability occur in low- and middle-income countries including Ghana. This 3-year retrospective review aimed to define, for the first time, the characteristics and outcomes of neonatal surgical conditions in northern Ghana.

**Methods:**

A retrospective study was conducted to include all admissions to the Tamale Teaching Hospital (TTH) neonatal intensive care unit (NICU) with surgical conditions between January 2014 and January 2017. Data were collected on demographics, diagnosis and outcomes. Descriptive analysis was performed on all data, and logistic regression was used to predict determinants of neonatal mortality. *p *< 0.05 was deemed significant.

**Results:**

Three hundred and forty-seven neonates were included. Two hundred and sixty-one (75.2%) were aged 7 days or less at presentation, with males (*n *= 177, 52%) slightly higher than females (*n *= 165, 48%). The majority were delivered by spontaneous vaginal delivery (*n *= 247, 88%); 191 (58%) were born in hospital. Congenital anomalies accounted for 302 (87%) of the neonatal surgical cases and 45 (96%) deaths. The most common anomalies were omphalocele (*n *= 48, 13.8%), imperforate anus (*n *= 34, 9.8%), intestinal obstruction (*n *= 29, 8.4%), spina bifida (*n *= 26, 7.5%) and hydrocephalus (*n *= 19, 5.5%). The overall mortality rate was 13.5%. Two-thirds of the deaths (*n *= 30) from congenital anomalies were conditions involving the digestive system with gastroschisis having the highest mortality of 88%. Omphalocele (*n *= 11, 23.4%), gastroschisis (*n *= 7, 14.9%) and imperforate anus (*n *= 6, 12.8%) contributed to the most deaths. On multivariate analysis, low birthweight was significantly associated with mortality (OR 3.59, CI 1.4–9.5, *p *= 0.009).

**Conclusion:**

Congenital anomalies are a major global health problem associated with high neonatal mortality in Ghana. The highest burden in terms of both caseload and mortality is attributed to congenital anomalies involving the digestive system, which should be targeted to improve outcomes.

**Electronic supplementary material:**

The online version of this article (10.1007/s00268-019-05210-9) contains supplementary material, which is available to authorized users.

## Introduction

In 2015, over 2 million neonates died worldwide [1]. Of these, an estimated 303,000 newborns died within 4 weeks of birth due to congenital anomalies [2]. Congenital anomalies, also known as congenital disorders, congenital malformations or birth defects, include abnormalities in a newborn’s structure, function or metabolism that lead to physical or mental disabilities. The World Health Organization (WHO) indicates that between 17 and 43% of infant mortality can be attributed to congenital anomalies [3]. Many life-saving, cost-effective treatments are available for congenital anomalies that can improve long-term outcomes [4, 5]. Surgery is an important, but largely neglected, component of the services available to treat congenital anomalies particularly in low- and middle-income countries (LMICs) [4].

LMICs have disproportionally high morbidity and mortality rates associated with congenital anomalies. Currently, it is estimated that 94% of congenital anomalies occur in LMICs [6]. The incidence of congenital anomalies in LMICs is between 3.9 and 11.8 per 1000 live births [7]. Mortality rates associated with congenital anomalies in LMICs range from 20 to 85% [6, 8]. Because half of all congenital anomalies are surgical, early treatment through the provision of surgical care is an obvious method to decrease the burden of childhood disease attributed to congenital anomalies [7, 9–11].

Ghana is a LMIC in sub-Saharan Africa (SSA) with a high mortality for children under 5 years at 56 per 1000 live births [12]. This is higher than the Sustainable Development Goal (SDG) for under-5 mortality of 37.0 per 1000 live births [13]. Factors such as poverty, lack of access to diagnostic procedures and timely treatment of congenital anomalies and other surgical conditions in neonates contribute to the high mortality rate amongst neonates in LMICs [14]. This retrospective 3-year study aimed to determine the pattern of neonatal surgical conditions in Ghana, identify factors which affect neonatal outcomes in these patients and to provide recommendations which will result in decreased mortality rates for such patients in the three northern regions of Ghana. This study is the first of its kind to be conducted in the Northern part of the country and will provide valuable information for both clinicians and policymakers alike, during planning for surgical conditions.

## Materials and methods

This retrospective study was conducted in the Tamale Teaching Hospital (TTH), a tertiary hospital located in the Northern Region of Ghana. The TTH is affiliated with the University for Development Studies, School of Medicine and Health Sciences. Patients are referred to TTH from primary and secondary health institutions from the Northern, Upper East and Upper West Regions of Ghana and sometimes from Burkina Faso, Cote d’Ivoire and Togo. The estimated total populations of these three regions are 2,479,461 (Northern Region: 59% of catchment area), 1,046,545 (Upper East Region: 25% of catchment area) and 702,110 (Upper West Region: 17% of catchment area) [15].

All neonates admitted to the neonatal intensive care unit (NICU) of the TTH with a surgical condition between January 2014 and January 2017 were included in the study. The NICU is a 40-bed capacity unit managed by one paediatrician, one medical officer, 4–6 house officers rotating at a time, two paediatric nurses and 34 general nurses. It is equipped with eight incubators, one radiant warmer, five cardio-respiratory monitors and four phototherapy machines. Oxygen supply is assured with oxygen concentrators (4) and oxygen cylinders. The unit is able to provide bubble CPAP therapy for babies who fail intranasal oxygen therapy. Surgical services are provided by paediatric surgeons (2), general surgeons (2), orthopaedic surgeons (2), neurosurgeon (1), ENT surgeons (3), and most surgical neonates are managed postoperatively in the NICU.

Data collected from hospital case files included patients’ age; gender; parents place of residence; place of birth and birthweight; gestation period and mode of delivery; diagnosis; referring hospital; length of stay in hospital; and mortality. Children were excluded if they were over the age of 28 days or if the age was unknown (*n* = 7). After the data were cleaned to exclude incomplete or anomalous data, the data were analysed using SAS 9.4 (SAS Institute Inc., Cary, NC). Descriptive analysis was performed on all data. Logistic regression was used to predict determinants of neonatal mortality. The independent variables included patient age, sex, mode of delivery, place of birth, and parents’ place of residence, gestation period and diagnosis.

## Results

### Patients

**Fig. 1:**
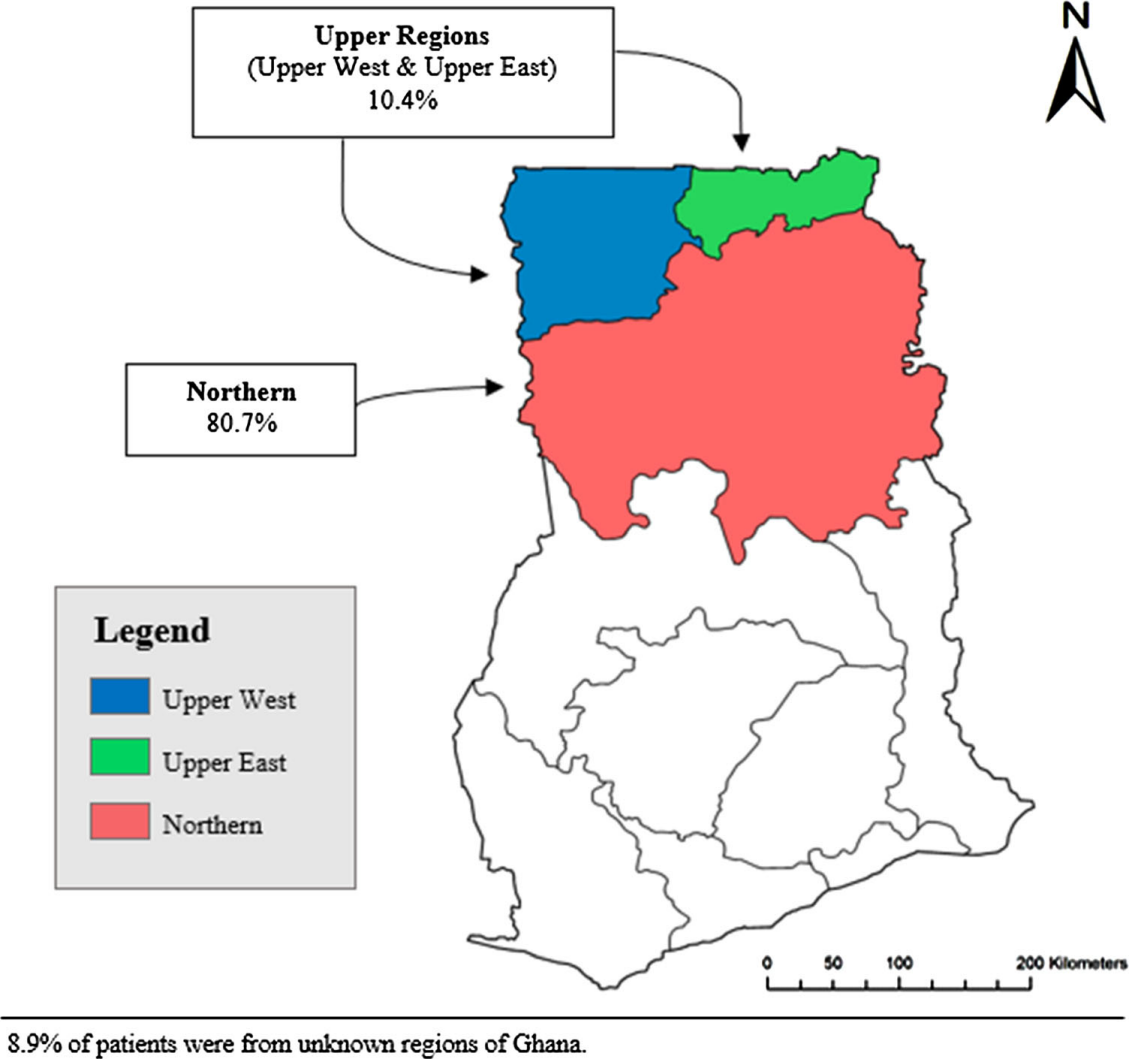
Map of Ghana showing the distribution of surgical neonatal cases from the three northern regions

Over the 3-year study period, 347 children aged 28 days or less were included in the study. These patients came from the three northern regions of Ghana, with 280 (80.7%) from the Northern Region and 36 (10.4%) from the Upper East and Upper West Regions; the regions of origin of 31 cases (8.9%) were unknown (Table 1 and Fig. 1). Neonates aged 7 days or younger represented the majority (*n* = 261, 75.2%) of the total cohort. There were 177 (51.0%) males and 165 (47.6%) females, with five cases (1.4%) being of unknown gender and a male-to-female ratio of 1.1:1.0. More neonates were born in the hospital (*n* = 191, 55%) than at home (*n* = 139, 40.1%); 17 (4.9%) unknown. The majority (*n* = 247, 88.2%) of patients with known method of delivery were born via spontaneous vaginal delivery. Most of the gestation periods were term deliveries greater than or equal to 38 weeks (*n* = 341, 98.3%). Of those with a known birthweight (*n* = 211), 72 (34.1%) were under 2500 g. Most patients had a hospital stay of 0–3 days (*n* = 147, 42.4%). Congenital anomalies accounted for 302 (87%) of all neonatal surgical cases. Congenital conditions involving the digestive system accounted for the majority of congenital anomalies (*n* = 144, 41.8%), followed by conditions of the nervous system (*n* = 63, 18.2%) and the musculoskeletal system (*n* = 37, 10.7%). Irrespective of the condition, most patients (*n* = 256, 73.8%) were discharged or died by day 7 post-admission. Supplemental Table 1 details the characteristics of neonatal surgical conditions by congenital malformation classification.

**Table 1:** Demographic characteristics of neonatal surgical patients in Ghana (*N *= 347)

	Total	Died	Survived	p*
	*n* (%)	*n* (%)	*n* (%)	
	347	13.5 (47)	86.5 (300)	
*Age (days)*				
0–7	75.2 (261)	85.1 (40)	73.7 (221)	
8–14	13.0 (45)	8.5 (4)	13.7 (41)	
15–21	8.9 (31)	4.3 (2)	9.7 (29)	0.1235
22–28	2.9 (10)	2.1 (1)	3.0 (9)	
*Sex*				
Male	51.0 (177)	57.5 (27)	50.0 (150)	0.4226
Female	47.6 (165)	40.4 (19)	48.7 (146)	
Unknown	1.4 (5)	2.1 (1)	1.3 (4)	
*Geographic region*				
Northern	80.7 (280)	68.1 (32)	82.7 (248)	
Upper regions (Upper East & Upper West)	10.4 (36)	17.0 (8)	9.3 (28)	0.9083
Unknown	8.9 (31)	14.9 (7)	8.0 (24)	
*Place of birth*				
Home	40.1 (139)	42.5 (20)	39.7 (119)	
Hospital	55.0 (191)	53.2 (25)	55.3 (166)	0.8802
Unknown	4.9 (17)	4.3 (2)	5.0 (15)	
*Mode of delivery*				
C-section	9.5 (33)	6.4 (3)	10.0 (30)	
Spontaneous vaginal delivery	71.2 (247)	63.8 (30)	72.3 (247)	0.0997
Unknown	19.3 (67)	29.8 (14)	17.7 (53)	
*Gestation*				
Preterm	1.1 (4)	4.3 (2)	0.6 (2)	
Term	98.3 (341)	95.7 (45)	98.7 (296)	0.4366
Unknown	0.6 (2)	0.0 (0)	0.7 (2)	
*LOS (days)*				
0–3	42.4 (147)	55.3 (26)	40.3 (121)	
4–7	31.4 (109)	23.4 (11)	32.7 (98)	
8–11	11.2 (39)	6.4 (3)	12.0 (36)	
12–15	5.5 (19)	6.4 (3)	5.3 (16)	0.0184
>15	4.6 (16)	0.0 (0)	5.3 (16)	
Unknown	4.9 (17)	8.5 (4)	4.3 (13)	
*Birthweight (g)*				
Normal BW (≥2500)	40.1 (139)	25.5 (12)	42.3 (127)	
LBW (<2500)	20.7 (72)	36.2 (17)	18.3 (55)	0.2596
Unknown	39.2 (136)	38.3 (18)	39.3 (118)	

Two patients did not have mortality outcome data and therefore were excluded from all analyses

*LOS* length of stay, *BW* birthweight, *LBW* low birthweight; term pregnancy is  ≥38 weeks; congenital malformation classifications are based on ICD-10 classifications

*p value excluded any missing or unknown data in the calculation

### Mortality

**Fig. 2:**
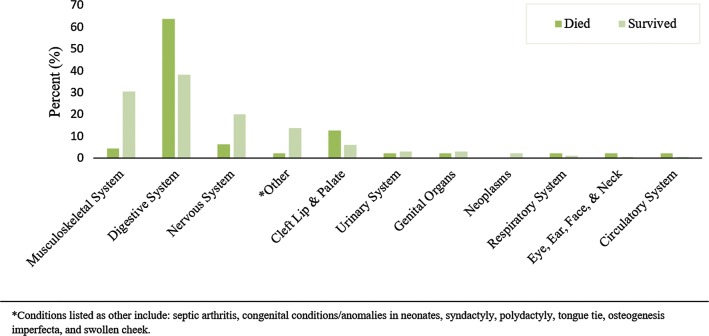
Proportion of deaths attributable to each congenital anomaly category

The overall mortality rate for neonates was 13.5% (Table 2). Neonates presenting between 0 and 7 days of life represented the majority of all deaths (*n* = 40, 85.1%), followed by neonates aged 8–14 days (*n* = 4, 8.5%) (Table 1). Amongst neonates presenting within 7 days of life, 40 (15.3%) died compared to seven (8.1%) of those presenting after 7 days. Mortality was marginally higher in males (27/177, 15%) compared to females (19/165, 12%). Congenital anomalies involving the digestive system accounted for 30 of the 47 deaths (63.8%) (Table 2 and Fig. 2). Omphalocele (*n* = 11, 23.4%), gastroschisis (*n* = 7, 14.9%) and imperforate anus (*n* = 6, 12.8%) contributed to the most deaths. Overall 30/144 (20.7%) died in the group with congenital anomalies involving the digestive system.

**Table 2:** Mortality by congenital anomaly classification

	Total (*n *= 347)	Died (*n *= 47)	Discharged/transferred (*n *= 300)
	*n*	*n* (%)	*n* (%)
*Musculoskeletal system*			
Talipes equinovarus	10	1 (10.0)	9 (90.0)
Prune belly syndrome	6	–	6 (100.0)
Femur fracture	6	–	6 (100.0)
Humeral fracture	4	–	4 (100.0)
Other	11	1 (9.1)	10 (90.9)
System total	37	2 (5.4)	35 (94.6)
*Digestive system*			
Omphalocele	48	11 (22.9)	37 (77.1)
Imperforate anus	34	6 (17.6)	28 (82.4)
Intestinal obstruction	29	3 (10.3)	26 (89.7)
Hirschsprung’s disease	17	2 (11.8)	15 (88.2)
Gastroschisis	8	7 (87.5)	1 (12.5)
Umbilical cord herniation	3	–	3 (100.0)
Other	5	1 (20.0)	4 (80.0)
System total	144	30 (20.8)	114 (79.2)
*Nervous system*			
Spina bifida	26	–	26 (100.0)
Hydrocephalus	19	2 (10.5)	17 (89.5)
Encephalocele	8	–	8 (100.0)
Meningocele	7	–	7 (100.0)
Myelomeningocele	2	–	2 (100.0)
Erb’s palsy	1	1 (100.0)	–
System total	63	3 (4.8)	60 (95.2)
*Cleft lip and palate*			
Cleft lip and palate	15	4 (26.7)	11 (73.3)
Cleft palate	5	–	5 (100.0)
Cleft lip	4	2 (50.0)	2 (50.0)
System total	24	6 (25.0)	18 (75.0)
*Urinary system*			
Bladder exstrophy	4	–	4 (100.0)
Posterior urethral valve	4	–	4 (100.0)
Other	4	2 (50.0)	2 (50.0)
System total	12	2 (16.7)	10 (83.3)
*Genital organs*			
Undescended testis	2	1 (50.0)	1 (50.0)
Hypospadias	2	–	2 (100.0)
Hydrocele	2	–	2 (100.0)
Rectovaginal fistula	2	–	2 (100.0)
Other	2	–	2 (100.0)
System total	10	1 (10.0)	9 (90.0)
*Neoplasms*			
Hemangioma	3	–	3 (100.0)
Other	2	–	2 (100.0)
System total	5	0	5 (100.0)
*Respiratory system*			
Laryngotracheomalacia	2	1 (50.0)	1 (50.0)
Laryngomalacia	2	–	2 (100.0)
System total	4	1 (25.0)	3 (75.0)
*Eye, ear, face and neck*			
Ectropion	2	1 (50.0)	1 (50.0)
Left anophthalmia	1	–	1 (100.0)
System total	3	1 (33.3)	2 (66.7)
*Circulatory system*			
Congenital heart defect	1	1 (100.0)	0
*Other*			
Breast abscess	22	–	22 (100.0)
Septic arthritis	6	1 (16.7)	5 (83.3)
Cephalohematoma	4	–	4 (100.0)
Mastitis	2	–	2 (100.0)
Other^a^	10	–	1 (100.0)
System total	44	1 (2.3)	43 (97.7)
*Total*	*347*	*47 (13.5)*	*300 (86.5)*

^a^Includes syndactyly, polydactyly, tongue tie, osteogenesis imperfecta, injection abscess, swollen cheek, cellulitis, wrist abscess

Mortality amongst patients presenting from the Northern region, where TTH is based, was lower (32/280, 11%) compared to patients presenting from the Upper East and Upper West regions (8/36, 22%). Mortality was similar amongst neonates born at home (20/139, 14%) and in hospital (25/191, 13%). Neonates born via vaginal delivery had a higher mortality (30/247, 12%) compared to those born via caesarean section (3/33, 9%). Of the 341 neonates born after term pregnancies, 45 died (13.2%); two (50%) of the four preterm neonates died. Mortality was highest in neonates who stayed just 0–3 days in hospital (26/147, 18%), followed by those who stayed for 4–7 days (11/109, 10%) and those who stayed over 8 days (6/74, 8%). The survival rate of neonates was directly proportional to the length of hospital stay (*p *= 0.02).

Of neonates with recorded birthweight (*n* = 211), there was a higher mortality amongst those with low birthweight (17/72, 24%) compared to those with normal birthweights (12/139, 9%). Low birthweight (<2500 g) was the most significant predictor of mortality in the univariate and multivariate regression analyses (Table 3). The odds of dying amongst neonates with low birthweight were 3.6 times higher than amongst neonates with normal birthweights, adjusting for other covariates (*p* = 0.009). No other variables were significant in the multivariate model.

**Table 3:** Univariate and multivariate regression results

Variables	Univariate regression		Multivariate regression	
	OR (95% CI)	*p* value	OR (95% CI)	*p* value
*Sex*				
Male	REF			
Female	1.4 (0.7, 2.6)	0.3126		
*Age (days)*				
0–7	REF		REF	
>7	0.5 (0.2, 1.1)	0.0968	0.5 (0.1, 1.9)	0.32
*Region*				
Northern	REF		REF	
Upper regions	2.2 (0.9, 5.3)	0.0726	2.3 (0.4, 10.2)	0.26
*Hospital referral*				
Within Tamale district	REF			
Outside Tamale district	1.1 (0.5, 2.2)	0.8118		
*Gestation*				
Term	REF			
Preterm	6.6 (0.9, 47.9)	0.0629		
*Birthweight*				
NBW	REF		REF	
LBW	3.3 (1.5, 7.3)*	0.0039	3.59 (1.4, 9.5)	0.009
*Mode of delivery*				
SVD	REF			
CS	0.7 (0.2, 2.5)	0.6110		
*POB*				
Hospital	REF			
Home	1.1 (0.6, 2.1)	0.7342		
*LOS*				
0–7	REF			
>7	0.5 (0.2, 1.3)	0.1592		

*REF* reference value, *NBW* normal birthweight, ≥2500 g, *LBW* low birthweight, <2500 g, *CS* C-section, *SVD* spontaneous vaginal delivery, *POB* place of birth, *LOS* length of stay; term pregnancy is ≥38 weeks gestation

*Significant value

## Discussion

Neonates with congenital anomalies present a challenge worldwide [16–23]. The stress of adjusting to postnatal life with poorly developed anatomical, physiological, metabolic and immunological functions makes the newborn a unique patient [17, 24–30]. These factors along with the presence of congenital anomalies and other conditions requiring surgical intervention make neonates high-risk surgical patients who require specialized care [17, 31–34]. This retrospective hospital-based study of neonates with surgical conditions found survival disparities depending on the type of condition and the infant’s birthweight.

The overall mortality in this study was 13.5%. This may be an underestimate of the true mortality as all-cause, in-hospital mortality was used and some cases were referred out and may not have survived such as with gastroschisis. Despite this, the survival rate documented is still higher than previously reported in the subregion [16]. The availability of resources for preoperative and postoperative care in the NICU and the wide range of surgical expertise in our hospital could have contributed to the better survival in our cohort.

Congenital anomalies involving the digestive system accounted for two-thirds of deaths. Omphalocele was the most frequent anomaly within this category, accounting for 48 cases with a 77% survival. Gastroschisis cases presented less frequently, but the condition is more difficult to manage in our setting and hence has a lower survival of just 12%. These findings are similar to those of Askarpour et al. [35] who also reported a higher prevalence of omphalocele than gastroschisis at their hospital, but a higher mortality rate from neonates undergoing surgery for gastroschisis than omphalocele.

Congenital anomalies involving the nervous system were the second most common anomalies encountered in our cohort, the majority of them being neural tube defects (43/63, 68.3%) and hydrocephalus (19/63, 30.2%). This has been previously documented [36]. The in-hospital mortality for this group was, however, lower than our overall mortality (6% vs. 13.5%), possibly due to the fact that most of these patients presented without acute complications and were operated beyond the newborn period after discharge from our NICU. This is unlike the anomalies involving the gastrointestinal tract which commonly require emergency neonatal surgery to avoid adverse outcomes.

Delayed access to care resulting in poor clinical condition on arrival and lack of neonatal surgical and anaesthetic capacity may have contributed to the higher mortality rates amongst neonates with congenital anomalies involving the digestive system; these problems have also been noted in other LMICs [37, 38]. We were not able to explore these factors further due to the retrospective nature of our study, but we did find that term babies with normal birthweight who were born in Northern Region (where our NICU is located) had higher chances of survival.

Birthweight was the most significant predictor of mortality in our study. Neonates who were categorized as low birthweight (<2500 g) had three times the odds of dying compared to those categorized as having normal birthweight. Previous studies have also identified low birthweight to be a risk factor in neonatal deaths; a multiregional study of LMICs found that approximately 54% of the observed deaths occurred in babies that weighed less than 2,500 g [5]. Similarly, it is estimated that preterm birth, which generally results in lower neonatal birthweight, is attributed to nearly 30% of all neonatal deaths worldwide [39]. In this study, neonates who were not carried to term had six times the odds of dying compared to those who were carried to term. Regardless of congenital anomaly, preterm and low-birthweight neonates often require highly sophisticated care, which is limited in our setting due to deficiencies in both staffing levels and facilities. For example, our NICU does not have the provision to provide ventilation or parenteral nutrition for neonates.

Neonates from outside the Northern Region, where TTH is located, had two times the odds of dying compared to those from the Northern Region of Ghana. These results may indicate that although inadequate care may be a potential cause of increased mortality, delayed care due to inadequate transportation and poor road networks may also exacerbate this problem [40]. Delayed care may also result from traditional beliefs to seek homeopathic care first rather than medical treatment [40].

These three northern regions of Ghana account for the highest infant and childhood mortalities from congenital anomalies [15]. Ghana’s infant mortality rate is approximately 41 per 1000 live births [15]. However, the northern regions surpass that estimate at 53.0 deaths per 1000 live births [15]. This is higher than the infant mortality rate of 51.5 per 1000 live births in SSA and the global rate of 30.3 deaths per 1000 live births [41]. In rural areas of Northern, Upper East and Upper West Regions, lack of healthcare infrastructure could delay treatment and positive surgical outcomes of congenital anomalies. Available data in these regions report lower numbers of both physicians and hospital beds for adults compared to the urban areas of the rest of Ghana [12]. Therefore, it is expected that the capacity to surgically treat neonates is even lower.

This is the first study from northern Ghana regarding neonatal surgical outcomes and highlights the need for enhanced efforts to improve outcomes within this population. Our hospital is currently participating in a multicentre clinical interventional study aimed at improving survival from neonates born with gastroschisis in low-resource settings [42]. This has the potential of improving the quality of care provided for these patients. The study involves outreach teaching from TTH to the surrounding regional and district hospitals and the creation of referral networks, which may also benefit other neonatal surgical conditions. Similarly, the study involves the development of some neonatal care protocols, such as for the provision of intravenous nutrition, and generic neonatal nursing training at TTH, which may benefit a wider range of neonates.

Investing in surgical care has been highlighted as integral to strengthen health systems and protect families from catastrophic expenditures related to surgical care and would reduce preventable death [43]. Investment in the establishment of a NICU in Tamale Teaching Hospital has improved survival rate of neonates in the northern region compared to neighbouring regions without a NICU. The implications of this study should be increasing surgical capacity and early interventions to treat these congenital anomalies, especially those involving the digestive system which result in the majority of early deaths.

A notable limitation of the study is the relatively small sample size, which reduces the statistical power and generalisability of our results and the presence of missing data. Another limitation was the fact that we were unable to assess the contribution of sepsis and other causes and contributors of death in our cohort. Future studies should aim to collect multicentre and multiregional hospital data and assess the contribution of sepsis and other determinants, like access, timing and availability of care, to survival in this setting. Future studies may also benefit from the categorisation and assessment of anomaly severity to examine differences in survival rates by condition severity and also evaluate how varying management strategies influence outcomes.

## Conclusion

Congenital anomalies are a global health concern and are related to a high mortality, especially in LMICs. Mortality is highest in the first week of life and is associated with low birthweight. Congenital anomalies involving the digestive system account for the highest caseload and highest mortality. Early access to a facility that can provide effective neonatal surgical care may reduce the possibility of dying from these congenital conditions. Investment in neonatal surgical care is vital to improve neonatal survival rates in Northern Ghana. This study highlights the urgent need for further research into the burden of congenital anomalies in LMICs globally and implementation of innovative solutions to improve outcomes in lower resource settings.

## Electronic supplementary material

Below is the link to the electronic supplementary material.

Supplementary material 1 (DOCX 37 kb)
