# Supplementary material for: Burden of Neonatal Surgical Conditions in Northern Ghana
Source: World J Surg. 2019 Oct 3;44(1):3–11. doi: 10.1007/s00268-019-05210-9 (PMC6925064; doi:10.1007/s00268-019-05210-9)
Supplement: Supplementary file 1 — Supplementary material 1 (DOCX 37 kb) [file 268_2019_5210_MOESM1_ESM.docx]

| **Burden of Neonatal Surgical Conditions in Northern Ghana**  **Supplemental Table 1: Characteristics of Neonatal Surgical Patients in Ghana by Congenital Malformation Classification (N=347)** | | | | | | | | | | | | | | | | | | | | | | | | |
| --- | --- | --- | --- | --- | --- | --- | --- | --- | --- | --- | --- | --- | --- | --- | --- | --- | --- | --- | --- | --- | --- | --- | --- | --- |
|  | **Total** | **Musculoskeletal system** | | **Digestive system** | | **Nervous system** | | **Other** | | **Cleft lip & palate** | | **Urinary system** | | **Genital organs** | | **Neoplasms** | | **Respiratory system** | | **Eye, ear, face, & neck** | | **Circulatory system** | | |
|  | % (n) | % (n) | | % (n) | | % (n) | | % (n) | | % (n) | | % (n) | | % (n) | | % (n) | | % (n) | | % (n) | | % (n) | | |
|  | 347 | 10.7 (37) | | 41.5 (144) | | 18.2 (63) | | 12.7 (44) | | 6.9 (24) | | 3.5 (12) | | 2.9 (10) | | 1.4 (5) | | 1.2 (4) | | 0.9 (3) | | 0.3 (1) | | |
|  |  | **D** | **S** | **D** | **S** | **D** | **S** | **D** | **S** | **D** | **S** | **D** | **S** | **D** | **S** | **D** | **S** | **D** | **S** | **D** | **S** | **D** | **S** |  |
|  |  | % (n) | % (n) | % (n) | % (n) | % (n) | % (n) | % (n) | % (n) | % (n) | % (n) | % (n) | % (n) | % (n) | % (n) | %(n) | % (n) | %(n) | % (n) | % (n) | % (n) | % (n) | % (n) |  |
|  |  | 5.4(2) | 94.6(35) | 20.8(30) | 79.2(114) | 4.7(3) | 95.2(60) | 2.3(1) | 97.7(43) | 25.0(6) | 75.0(18) | 8.3(1) | 91.7(11) | 10.0(1) | 90.0(9) | 0.0(0) | 100.0(5) | 25.0(1) | 75.0(3) | 33.3(1) | 66.7(2) | 100.0(1) | 0.0(0) |  |
| Age (days) |  |  |  |  |  |  |  |  |  |  |  |  |  |  |  |  |  |  |  |  |  |  |  |  |
| 0-7 | 75.2(261) | 100.0(2) | 83.3(29) | 90.0(27) | 75.4(86) |  | 93.3(56) | 0(0) | 30.3(13) | 83.3(5) | 100.0(18) | 100.0(1) | 72.7(8) | 100.0(1) | 77.8(7) | 0.0(0) | 40.0(2) | 0.0(0) | 0.0(0) | 100.0(0) | 100.0(2) | 0.0(0) | 0.0(0) |  |
| 7-14 | 13.0(45) | 0.0(0) | 10.8(4) | 3.3(1) | 8.8(10) |  | 5.0(3) | 0(0) | 44.2(19) | 16.7(1) | 0.0(0) | 0.0(0) | 27.3(3) | 0.0(0) | 11.1(1) | 0.0(0) | 0.0(0) | 100.0(1) | 33.3(1) | 0.0(0) | 0.0(0) | 100.0(1) | 0.0(0) |  |
| 15-21 | 8.9(31) | 0.0(0) | 5.6(2) | 3.3(1) | 12.3(14) |  | 1.7(1) | 100.0(1) | 18.6(8) | 0.0(0) | 0.0(0) | 0.0(0) | 0.0(0) | 0.0(0) | 0.0(0) | 0.0(0) | 40.0(2) | 0.0(0) | 66.7(2) | 0.0(0) | 0.0(0) | 0.0(0) | 0.0(0) |  |
| 22-28 | 2.9(10) | 0.0(0) | 0.0(0) | 3.3(1) | 3.5(4) |  | 0.0(0) | 0.0(0) | 7.0(3) | 0.0(0) | 0.0(0) | 0.0(0) | 0.0(0) | 0.0(0) | 11.1(1) | 0.0(0) | 20.0(1) | 0.0(0) | 0.0(0) | 0.0(0) | 0.0(0) | 0.0(0) | 0.0(0) |  |
| Sex |  |  |  |  |  |  |  |  |  |  |  |  |  |  |  |  |  |  |  |  |  |  |  |  |
| Male | 51.0(177) | 50.0(1) | 55.6(20) | 53.3(16) | 57.0(65) | 100.0(3) | 45.0(27) | 0.0(0) | 39.5(17) | 66.7(4) | 16.7(3) | 100.0(1) | 45.5(5) | 100.0(1) | 66.7(6) | 0.0(0) | 40.0(2) | 100.0(1) | 100.0(3) | 0.0(0) | 100.0(2) | 0.0(0) | 0.0(0) |  |
| Female | 47.6(165) | 0.0(0) | 38.9(13) | 43.3(13) | 40.4(46) | 0.0(0) | 55.0(33) | 100.0(1) | 60.5(26) | 33.3(2) | 83.3(15) | 0.0(0) | 54.5(6) | 0.0(0) | 33.3(3) | 0.0(0) | 60.0(3) | 0.0(0) | 0.0(0) | 100.0(1) | 0.0(0) | 100.0(1) | 0.0(0) |  |
| Unknown | 1.4 (5) | 50.0(1) | 5.6(2) | 3.3(1) | 2.6(3) | 0.0(0) | 0.0(0) | 0.0(0) | 0.0(0) | 0.0(0) | 0.0(0) | 0.0(0) | 0.0(0) | 0.0(0) | 0.0(0) | 0.0(0) | 0.0(0) | 0.0(0) | 0.0(0) | 0.0(0) | 0.0(0) | 0.0(0) | 0.0(0) |  |
| Region |  |  |  |  |  |  |  |  |  |  |  |  |  |  |  |  |  |  |  |  |  |  |  |  |
| Northern | 80.7(280) | 50.0(1) | 83.3(30) | 73.3(22) | 85.7(98) | 66.67(2) | 71.67(43) | 0.0(0) | 86.05(37) | 50.0(3) | 77.78(14) | 100.0(1) | 90.91(10) | 100.0(1) | 100.0(9) | 0.0(0) | 60.0(3) | 100.0(1) | 66.67(2) | 100.0(1) | 100.0(2) | 0.0(0) | 0.0(0) |  |
| Upper Regions* | 10.4(36) | 0.0(0) | 5.6(2) | 20.0(6) | 6.1(7) | 0(0) | 16.67(10) | 0.0(0) | 6.98(3) | 16.67(1) | 16.67(3) | 0.0(0) | 9.09(1) | 0.0(0) | 0.0(0) | 0.0(0) | 20.0(1) | 0.0(0) | 33.33(1) | 0.0(0) | 0.0(0) | 100(1) | 0.0(0) |  |
| Unknown | 8.9(31) | 50.0(1) | 11.1(3) | 6.7(2) | 7.9(9) | 33.33(1) | 11.67(7) | 100.0(1) | 6.98(3) | 33.33(2) | 5.56(1) | 0.0(0) | 0.0(0) | 0.0(0) | 0.0(0) | 0.0(0) | 20.0(1) | 0.0(0) | 0.0(0) | 0.0(0) | 0.0(0) | 0.0(0) | 0.0(0) |  |
| Place of Birth |  |  |  |  |  |  |  |  |  |  |  |  |  |  |  |  |  |  |  |  |  |  |  |  |
| Home | 40.1(139) | 50.0(1) | 25.1(9) | 43.3(13) | 43.9(50) | 0.0(0) | 43.3(26) | 100.0(1) | 32.6(14) | 50.0(3) | 50.0(9) | 0.0(0) | 45.5(5) | 0.0(0) | 33.3(3) | 0.0(0) | 20.0(1) | 0.0(0) | 33.3(1) | 100.0(1) | 50.0(1) | 100.0(1) | 0.0(0) |  |
| Hospital | 55.0(191) | 0.0(0) | 65.7(23) | 56.7(17) | 50.9(58) | 100.0(3) | 50.0(30) | 0.0(0) | 62.8(27) | 50.0(3) | 50.0(9) | 100.0(1) | 54.5(6) | 0.0(0) | 66.7(6) | 0.0(0) | 80.0(4) | 100.0(1) | 66.7(2) | 0.0(0) | 50.0(1) | 0.0(0) | 0.0(0) |  |
| Unknown | 4.9(17) | 50.0(1) | 8.6(3) | 0.0(0) | 5.3(6) | 0.0(0) | 6.7(4) | 0.0(0) | 4.6(2) | 0.0(0) | 0.0(0) | 0.0(0) | 0.0(0) | 100.0(1) | 0.0(0) | 0.0(0) | 0.0(0) | 0.0(0) | 0.0(0) | 0.0(0) | 0.0(0) | 0.0(0) | 0.0(0) |  |
| Birthweight (gm) |  |  |  |  |  |  |  |  |  |  |  |  |  |  |  |  |  |  |  |  |  |  |  |  |
| Normal BW (≥2500) | 40.1(139) | 0.0(0) | 34.3(12) | 23.3(7) | 17.5(20) | 66.7(2) | 36.7(22) | 0.0(0) | 55.8(24) | 16.7(1) | 27.8(5) | 0.0(0) | 45.4(5) | 0.0(0) | 77.8(7) | 0.0(0) | 20.0(1) | 0.0(0) | 66.7(2) | 100.0(1) | 100.0(2) | 100.0(1) | 0.0(0) |  |
| LBW (<2500) | 20.7(72) | 50.0(1) | 22.9(8) | 36.7(11) | 41.2(47) | 0.0(0) | 28.3(17) | 100.0(1) | 7.0(3) | 50.0(3) | 22.2(4) | 0.0(0) | 9.2(1) | 100.0(1) | 0.0(0) | 0.0(0) | 40.0(2) | 0.0(0) | 0.0(0) | 0.0(0) | 0.0(0) | 0.0(0) | 0.0(0) |  |
| Unknown | 39.2(136) | 50.0(1) | 42.9(15) | 40.0(12) | 41.2(47) | 33.3(1) | 35.0(21) | 0.0(0) | 37.2(16) | 33.3(2) | 50.0(9) | 100.0(1) | 45.4(5) | 0.0(0) | 22.2(2) | 0.0(0) | 40.0(2) | 100.0(1) | 33.3(1) | 0.0(0) | 0.0(0) | 0.0(0) | 0.0(0) |  |
| Mode of Delivery |  |  |  |  |  |  |  |  |  |  |  |  |  |  |  |  |  |  |  |  |  |  |  |  |
| C-section | 9.5(33) | 0.0(0) | 14.3(5) | 3.3(1) | 4.4(5) | 66.7(2) | 18.3(11) | 0.0(0) | 13.9(6) | 0.0(0) | 0.0(0) | 0.0(0) | 9.1(1) | 0.0(0) | 22.2(2) | 0.0(0) | 0.0(0) | 0.0(0) | 0.0(0) | 0.0(0) | 0.0(0) | 0.0(0) | 0.0(0) |  |
| Spontaneous vaginal delivery | 71.2(247) | 0.0(0) | 68.6(24) | 76.7(23) | 74.6(85) | 33.3(1) | 65.0(39) | 0.0(0) | 69.8(30) | 33.3(2) | 83.3(15) | 100.0(1) | 81.8(9) | 0.0(0) | 66.7(6) | 0.0(0) | 80.0(4) | 100.0(1) | 100.0(3) | 100.0(1) | 100.0(2) | 100.0(1) | 0.0(0) |  |
| Unknown | 19.3(67) | 100.0(2) | 17.1(6) | 20.0(6) | 21.1(24) | 0.0(0) | 16.7(10) | 100.0(1) | 16.3(7) | 66.7(4) | 16.7(3) | 0.0(0) | 9.1(1) | 100.0(1) | 11.1(1) | 0.0(0) | 20.0(1) | 0.0(0) | 0.0(0) | 0.0(0) | 0.0(0) | 0.0(0) | 0.0(0) |  |
| Gestation |  |  |  |  |  |  |  |  |  |  |  |  |  |  |  |  |  |  |  |  |  |  |  |  |
| Preterm | 1.1(4) | 0.0(0) | 0.0(0) | 0.0(0) | 0.0(0) | 0.0(0) | 3.3(2) | 0.0(0) | 0.0(0) | 16.7(1) | 0.0(0) | 0.0(0) | 0.0(0) | 100.0(1) | 0.0(0) | 0.0(0) | 0.0(0) | 0.0(0) | 0.0(0) | 0.0(0) | 0.0(0) | 0.0(0) | 0.0(0) |  |
| Term | 98.3(341) | 100.0(2) | 100.0(35) | 100.0(30) | 98.2(112) | 100.0(3) | 95.0(57) | 100.0(1) | 100.0(43) | 83.3(5) | 100.0(18) | 100.0(1) | 100.0(11) | 0.0(0) | 100.0(9) | 0.0(0) | 100.0(5) | 100.0(1) | 100.0(3) | 100.0(1) | 100.0(2) | 100.0(1) | 0.0(0) |  |
| Unknown | 0.6(2) | 0.0(0) | 0.0(0) | 0.0(0) | 1.8(2) | 0.0(0) | 1.7(1) | 0.0(0) | 0.0(0) | 0.0(0) | 0.0(0) | 0.0(0) | 0.0(0) | 0.0(0) | 0.0(0) | 0.0(0) | 0.0(0) | 0.0(0) | 0.0(0) | 0.0(0) | 0.0(0) | 0.0(0) | 0.0(0) |  |
| LOS (days) |  |  |  |  |  |  |  |  |  |  |  |  |  |  |  |  |  |  |  |  |  |  |  |  |
| 0-3 | 42.4(147) | 100.0(2) | 54.3(19) | 70.0(21) | 36.8(42) | 33.3(1) | 35.0(21) | 100.0(1) | 41.9(18) | 16.7(1) | 38.9(7) | 100.0(1) | 45.4(5) | 0.0(0) | 55.6(5) | 0.0(0) | 20.0(1) | 0.0(0) | 33.3(1) | 0.0(0) | 50.0(1) | 0.0(0) | 0.0(0) |  |
| 4-7 | 31.4(109) | 0.0(0) | 31.4(11) | 20.0(6) | 33.3(38) | 33.3(1) | 28.3(17) | 0.0(0) | 37.2(16) | 33.3(2) | 44.4(8) | 0.0(0) | 9.1(1) | 100.0(1) | 33.3(3) | 0.0(0) | 40.0(2) | 100.0(1) | 66.7(2) | 0.0(0) | 0.0(0) | 0.0(0) | 0.0(0) |  |
| 8-11 | 11.2(39) | 0.0(0) | 8.6(3) | 3.3(1) | 12.3(14) | 0.0(0) | 15.0(9) | 0.0(0) | 9.3(4) | 33.3(2) | 5.6(1) | 0.0(0) | 36.4(4) | 0.0(0) | 0.0(0) | 0.0(0) | 20.0(1) | 0.0(0) | 0.0(0) | 0.0(0) | 0.0(0) | 0.0(0) | 0.0(0) |  |
| 12-15 | 5.5(19) | 0.0(0) | 5.7(2) | 3.3(1) | 5.3(6) | 33.3(1) | 10.0(6) | 0.0(0) | 4.6(2) | 0.0(0) | 0.0(0) | 0.0(0) | 0.0(0) | 0.0(0) | 0.0(0) | 0.0(0) | 0.0(0) | 0.0(0) | 0.0(0) | 0.0(0) | 0.0(0) | 100.0(1) | 0.0(0) |  |
| >15 Days | 4.6(16) | 0.0(0) | 0.0(0) | 0.0(0) | 7.0(8) | 0.0(0) | 6.7(4) | 0.0(0) | 4.6(2) | 0.0(0) | 0.0(0) | 0.0(0) | 9.1(1) | 0.0(0) | 0.0(0) | 0.0(0) | 20.0(1) | 0.0(0) | 0.0(0) | 0.0(0) | 0.0(0) | 0.0(0) | 0.0(0) |  |
| Unknown | 4.9(17) | 0.0(0) | 0.0(0) | 3.3(1) | 5.3(6) | 0.0(0) | 5.0(3) | 0.0(0) | 2.3(1) | 16.7(1) | 11.1(2) | 0.0(0) | 0.0(0) | 0.0(0) | 11.1(1) | 0.0(0) | 0.0(0) | 0.0(0) | 0.0(0) | 100.0(1) | 50.0(1) | 0.0(0) | 0.0(0) |  |
| D= Died; S=Survived; BW= Birthweight; LBW=Low birth weight; LOS= Length of stay; Term pregnancy is ≥38 weeks | | | | | | | | | | |  |  |  |  |  |  |  |  |  |  |  |  |  |  |
| *Upper Regions includes the Upper East and Upper West regions in Ghana | | | | | | |  |  |  |  |  |  |  |  |  |  |  |  |  |  |  |  |  |  |
